# Optimal *st*-PMMA/C_60_ helical inclusion complexes via tunable energy landscapes for the application of an Ag SERS-active substrate

**DOI:** 10.1107/S1600576725001712

**Published:** 2025-03-19

**Authors:** Song-Yu Tsai, Wen-Tsung Tseng, Jina-Hua Su, Yu-Hao Wang, Yi-Wei Chang, Chia-Hsin Wang, U-Ser Jeng, Kuan-Yi Wu, Chien-Lung Wang, Wei-Tsung Chuang

**Affiliations:** a Department of Applied Chemistry, National Yang Ming Chiao Tung University, 1001 Ta Hsueh Road, Hsinchu30010, Taiwan; b Department of Chemical Engineering and Biotechnology National Taipei University of Technology Taipei10608 Taiwan; c National Synchrotron Radiation Research Center 101 Hsin-Ann Road Hsinchu30076 Taiwan; d Department of Chemistry National Taiwan University No. 1, Sec. 4, Roosevelt Rd Taipei10631 Taiwan; Australian Centre for Neutron Scattering, ANSTO, Australia

**Keywords:** supramolecular chemistry, inclusion complexes, helical polymers, C_60_, Raman spectroscopy

## Abstract

In bio-inspired systems, the hierarchical structures of biomolecules are mimicked to impart desired functions to self-assembled materials. However, these hierarchical architectures are based on multicomponent systems, which require not only a well defined primary structure of functional molecules but also the programming of self-assembly pathways. In this study, we investigate pathway complexity in the energy landscape of the syndiotactic poly(methyl methacrylate) (*st*-PMMA)/C_60_/toluene complex system, where C_60_ and toluene serve as guests in the *st*-PMMA helical host. Structural characterization revealed that *st*-PMMA preferentially wraps around C_60_, forming a thermodynamically favorable helical inclusion complex (HIC). However, during the preparation of the *st*-PMMA/C_60_ HIC, a lengthy guest-exchange pathway was discovered, where the *st*-PMMA/toluene HIC transformed into the *st*-PMMA/C_60_ HIC. This pathway complexity may hinder the formation of the *st*-PMMA/C_60_ HIC within a feasible timeframe. Given that the energy landscape can be modulated by temperature, the *st*-PMMA host can directly wrap around C_60_ in higher temperature ranges, thereby bypassing the guest-exchange process and increasing the *st*-PMMA/C_60_ HIC formation efficiency. Additionally, after self-assembly programming, the *st*-PMMA/C_60_ HIC can serve as an excellent photochemical reduction site. The well dispersed nanodomains of the *st*-PMMA/C_60_ HICs act as nanoparticle templates for surface-enhanced Raman scattering (SERS) hotspot fabrication. We successfully utilized these HIC templates to synthesize self-assembled SERS-active silver nanoparticle arrays, demonstrating their potential for use in chemical sensing applications. In summary, a clear energy landscape can guide supramolecular engineering to achieve the desired supramolecular architectures by selecting appropriate self-assembly pathways.

## Abstract 2

This study tunes the energy landscape of the syndiotactic poly(methyl methacrylate) (*st*-PMMA)/C_60_/toluene complex system and finds the efficient pathway to form the thermodynamically favored *st*-PMMA/C_60_ complex by temperature modulation. This *st*-PMMA/C_60_ complex architecture further acts as a photoreduction site for yielding the Ag surface-enhanced Raman scattering (SERS)-active substrate for advanced chemical sensing applications.

## 1. Introduction

In nature, hierarchical architectures such as ribosomes, DNA and lipid membranes are formed through multicomponent self-assembly processes (Harayama & Riezman, 2018 ▸). To ensure the formation of complex structures, it is necessary to maintain the fidelity of molecular architecture such as chain length and sequence in polypeptides and DNA strands (Zaher & Green, 2009 ▸). Nature cleverly uses enzymes to realize accurate sequences in polynucleotides or polypeptide chains (Novacek *et al.*, 2024 ▸). However, providing the well defined molecular architecture of biomolecules alone does not guarantee the formation of these intricate complexes because self-assembly is pathway dependent (Knowles *et al.*, 2014 ▸). The hierarchical self-assembly of biomolecules is encoded in the collective contribution of various non-covalent interactions between bio-entities (*e.g.* salt bridges, hydro­phobic interactions, hydrogen-bond interactions) (Szilágyi & Závodszky, 2000 ▸). Although the competition among these non-covalent interactions further brings out several self-assembled states with local or global energy minima, living systems can navigate pathways within the energy landscape to guide biomolecules toward the desired supramolecular architectures with specific physiological functions (Adamcik & Mezzenga, 2018 ▸; Ma *et al.*, 2020 ▸; Nishimura & Akiyoshi, 2020 ▸).

Learning from nature, bio-inspired self-assembly usually attempts to mimic the definitive primary structure of bio­mol­ecules, for instance, chain length and sequence control. This approach opens the first step toward endowing synthetic molecules with structural complexity in supramolecular chemistry. For example, synthetic helical polymers are usually obtained by controlling stereoregularity, such as syndiotactic polystyrene and poly(acetyl­enes) (Yashima *et al.*, 2016 ▸). Among the synthetic stereoregular polymers, syndiotactic poly(methyl methacrylate) (*st*-PMMA) is a representative example that exhibits helical wrapping behavior in two-/multicomponent systems, similar to natural helical polymers such as DNA strands and polysaccharides (Yashima *et al.*, 2016 ▸; Zhang & Seelig, 2011 ▸; Fittolani *et al.*, 2020 ▸). *st*-PMMAs can form helical inclusion complexes (HICs) with specific guest molecules through molecular recognition, for example, aromatic solvents, polycyclic aromatic hydro­carbons, fullerenes and isotactic PMMA (*it*-PMMA) (Yashima *et al.*, 2016 ▸; Kawauchi *et al.*, 2010 ▸; Kawauchi *et al.*, 2011 ▸; Ren *et al.*, 2018 ▸). *st*-PMMA subsequently undergoes a conformational change to the helical structure, providing a cavity *ca* 1 nm in size, which allows for the inclusion of these guest molecules through the induced-fit mechanism (Kawauchi *et al.*, 2010 ▸; Kawauchi *et al.*, 2011 ▸; Ren *et al.*, 2018 ▸; Bosshard, 2001 ▸). Unlike DNA wrapping through complementary hydrogen-bond inter­actions, although *st*-PMMA complexation is driven by van der Waals forces, its binding selectivity to guest molecules can be still determined by various traits of its chemical structure such as size, chain length and stereoregularity (Kawauchi *et al.*, 2010 ▸; Ren *et al.*, 2018 ▸; Kajihara *et al.*, 2020 ▸). Thus, the unique self-assembly behavior of the *st*-PMMA multicomponent system extends its applications to sensing, separation and catalysis, in addition to electronic and opto­electronic materials (Qi *et al.*, 2013 ▸; Li *et al.*, 2022 ▸; Chen *et al.*, 2023 ▸).

In *st*-PMMA-based applications, the self-assembly process occurs in the multi-component system, where at least two types of guests can bind with the *st*-PMMA host (Yashima *et al.*, 2016 ▸; Kawauchi *et al.*, 2010 ▸; Kawauchi *et al.*, 2011 ▸). Competition among the guest molecules for binding may lead to multiple self-assembled states in the energy landscape, hindering structural control over the *st*-PMMA multicomponent system (Kawauchi *et al.*, 2011 ▸; Kawauchi *et al.*, 2008 ▸). For instance, during the formation of the *st*-PMMA/C_60_ HIC where toluene is used as the solvent, *st*-PMMA/toluene HICs are also formed in solution due to toluene acting as a guest of *st*-PMMA hosts. To control the complex architecture, conventional supramolecular strategies rely on precise synthesis to tailor the *st*-PMMA structure, for example, regulation of the *rr* content and molecular weight, which can improve the binding specificity of a specific guest (Ren *et al.*, 2018 ▸). Kajihara *et al.* (2020 ▸) tried to perfect the stereoregularity of the *st*-PMMA chain, but the amount of C_60_ encapsulated in the *st*-PMMA helix increased only marginally, and the encapsulation ratio was considerably lower than the ideal value of 28 wt%. By contrast, bio-systems allow for the formation of complex architectures by precisely controlling bio-entities, which entails guiding them to the correct pathway in the energy landscape (Knowles *et al.*, 2014 ▸; Ma *et al.*, 2020 ▸). This observation motivated us to explore the thermodynamic stability of each supramolecular species in the *st*-PMMA multicomponent system and explore the self-assembly pathways in its energy landscape.

Herein, we investigate the complexity of self-assembly pathways in the three-component *st*-PMMA/C_60_/toluene system, where C_60_ and toluene act as guests in the *st*-PMMA helical host, as illustrated in Scheme [Chem scheme1](*a*). The concentration- and temperature-dependent structural characterizations of *st*-PMMA-based HICs are firstly revealed through simultaneous small- and wide-angle X-ray scattering (SAXS and WAXS). Three self-assembled species are then identified in the *st*-PMMA/C_60_/toluene system: helical-rich *st*-PMMA clusters, *st*-PMMA/toluene HICs and *st*-PMMA/C_60_ HICs [Scheme [Chem scheme1](*b*)]. In terms of binding affinity, the tighter binding of *st*-PMMA and C_60_ indicates that the *st*-PMMA/C_60_ HIC is thermodynamically favorable. Next, we strategically apply temperature modulation to the energy landscape to accelerate the formation of *st*-PMMA/C_60_ HIC structures and increase the C_60_ encapsulation efficiency considerably. We find that programming the self-assembly pathway can lead to a high encapsulation ratio in *st*-PMMA/C_60_ HICs without the need for the time-consuming guest-exchange pathway. Furthermore, the resulting *st*-PMMA/C_60_ HICs exhibit well dispersed C_60_ domains and act as effective reduction templates for Ag nanoparticle (Ag-NP) synthesis. These Ag-NPs intensify surface-enhanced Raman scattering (SERS) activity and outperform Ag-decorated C_60_ crystals in rhodamine 6G detection. By programming self-assembly pathways, this approach optimizes C_60_ encapsulation and enhances Ag-NP production, thereby demonstrating the potential for synthesizing targeted supramolecular architectures for functional applications.
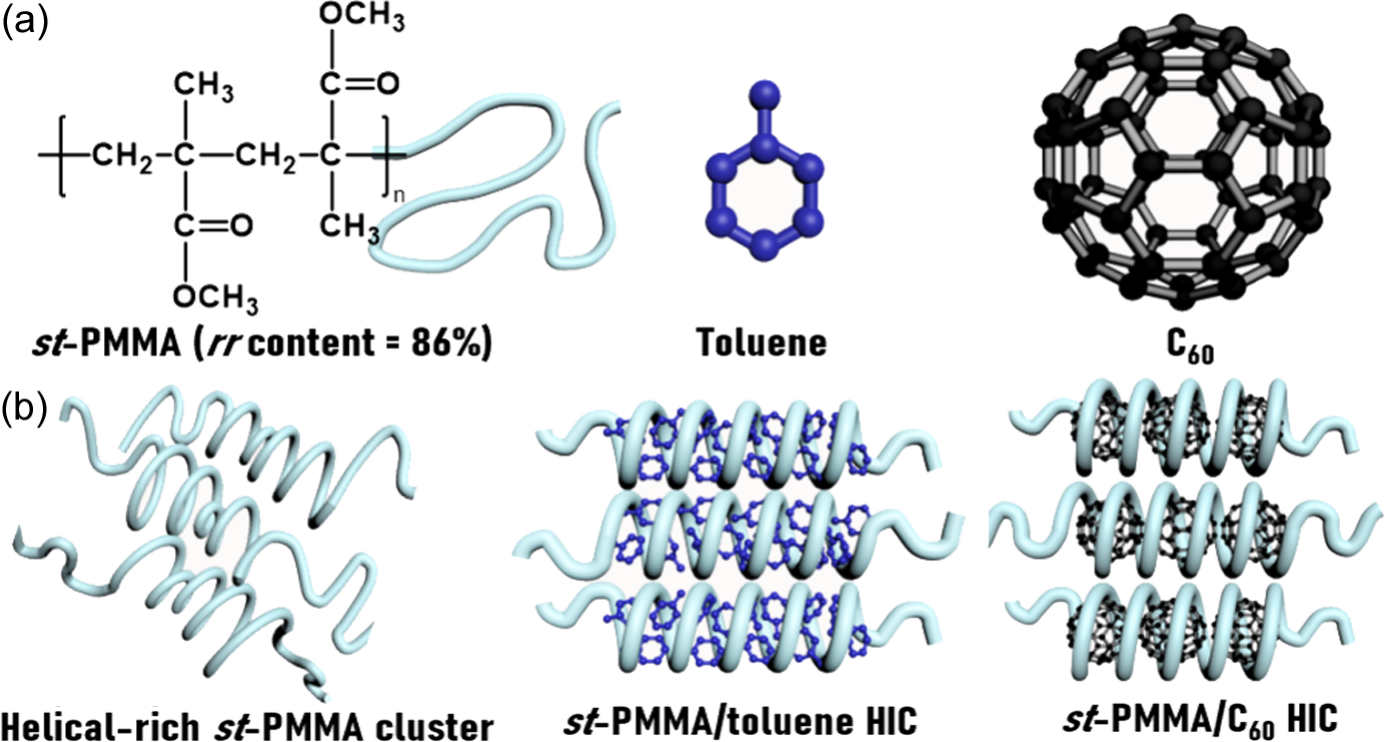


## 2. Experimental

### 2.1. Materials and methods

C_60_ (purity = 99.5%) and *st*-PMMA (number-average mol­ecular weight *M*_n_ = 31.2 kg mol^−1^ and *rr* content = 86%) were purchased from Alfa Aesar and Polymer Source Inc., respectively. All other reagents and solvents were purchased from Sigma–Aldrich and were used without purification. The *rr* content of the *st*-PMMA was measured using ^1^H nuclear magnetic resonance (NMR) spectrometry. NMR spectra were recorded using an Agilent Unity-400 NMR spectrometer, where CDCl_3_ was employed as a deuterated solvent to identify the molecular structures at 25°C. *M*_n_ and the polydispersity index of *st*-PMMA were determined using a gel permeation chromatograph equipped with a JASCO liquid chromatograph, comprising a JASCO PU-4180 pump, JASCO RI-4030 detector and Stragel columns (HR1, HR2 and HR4). Tetra­hydro­furan (THF) was utilized as the eluent at a flow rate of 1.0 ml min^−1^ and temperature of 30°C. The measurements were performed at 30°C.

### 2.2. Infrared spectroscopy

Attenuated total reflection (ATR)/Fourier transform-infrared (FT-IR) spectra of the *st*-PMMA/toluene and *st*-PMMA/C_60_/toluene solution/gel were obtained using a PerkinElmer Spectrum 3 spectrometer equipped with a ZnSe crystal ATR attachment. The IR spectra were recorded over 16 scans in the wavenumber range 920–780 cm^−1^. Synchrotron FT-IR spectroscopy measurements were performed at the endstation of the TLS14A beamline of the Taiwan Light Source at the National Synchrotron Radiation Research Center (NSRRC), Taiwan.

### 2.3. Simultaneous small-angle X-ray scattering/wide-angle X-ray scattering

Simultaneous SAXS/WAXS measurements were recorded using the TPS BL13A beamline at NSRRC. The scattering signals were collected using the Eiger X 9M and Eiger X 1M detectors. The wavelength of the X-rays was 0.827 Å. The scattering vector magnitude, *q*, related to the scattering angle (2θ) and photon wavelength (λ), was calculated using the equation *q* = 4π sin(θ)/λ. Samples were loaded into quartz capillary tubes and sealed by silicone resin. Finally, the tubes were mounted on the temperature-controlled stage to conduct the SAXS/WAXS measurements.

### 2.4. X-ray photoelectron spectroscopy

X-ray photoelectron spectroscopy (XPS) was performed at the endstation of the BL24A beamline at NSRRC. The XPS endstation is equipped with a load lock chamber for sample loading, an ultrahigh vacuum preparation chamber for sample preparation and a main analysis chamber for electron spectroscopy measurements, which are carried out using a SPECS NAP 150 electron energy analyzer.

### 2.5. Scanning electron microscopy

Scanning electron microscopy (SEM) analysis was performed using a JEOL JSM-7610F microscope operating at an accelerating voltage of 5 kV.

### 2.6. Raman spectroscopy

Raman spectroscopy was performed using a laboratory-built micro-Raman system. A Cobolt 532 samba laser was used to irradiate the samples and scattering signals were collected using a Southport Jademat NM system equipped with a Kymera 328i B1 Andor spectrometer. The laser exposure time was set to 3 s for each spectrum.

## 3. Result and discussion

### 3.1. Solvent effect on *st*-PMMA HICs

**Figure 1:**
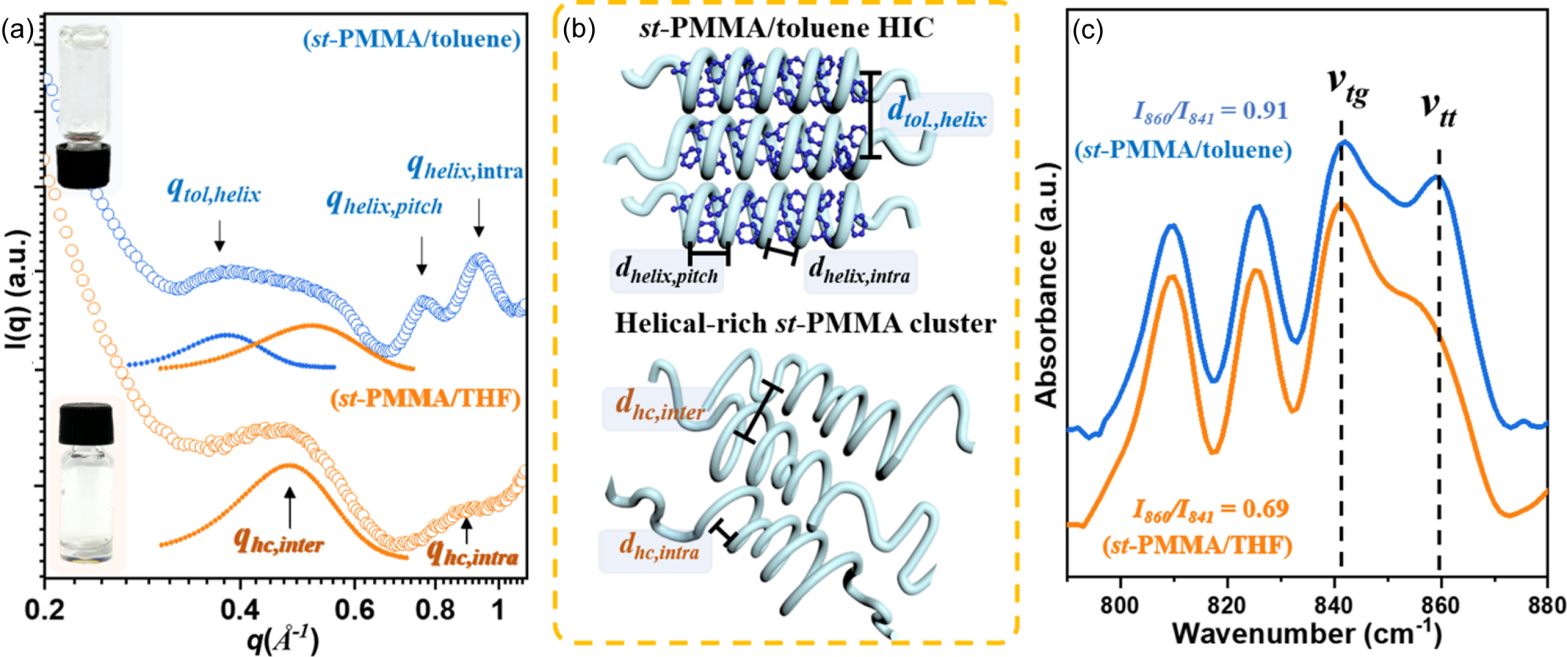
(*a*) Inverted vial test photographs and WAXS profiles of the *st*-PMMA/toluene gel and *st*-PMMA/THF solution at [*st*-PMMA] = 0.4 *M*. (*b*) Structural illustration of the *st*-PMMA/toluene HIC and helical-rich *st*-PMMA cluster. (*c*) IR spectra of the *st*-PMMA/toluene gel and *st*-PMMA/THF solution at [*st*-PMMA] = 0.4 *M*.

Molecular characterization results of the *st*-PMMA host with *M*_n_ = 31 kg mol^−1^ and *rr* content = 86% are presented in Figs. S1 and S2 of the supporting information. To investigate the solvent effect on the *st*-PMMA self-assembly, THF and toluene were used as solvents to prepare the *st*-PMMA solutions at [*st*-PMMA] = 0.4 *M* (80 mg ml^−1^). The *st*-PMMA exhibited better solubility in THF than in toluene (Minei *et al.*, 2014 ▸). As illustrated in the inset of Fig. 1 ▸(*a*), the *st*-PMMA/THF system formed a solution, but the *st*-PMMA/toluene system was in a gel state. Fig. 1 ▸(*a*) presents the WAXS profiles of the *st*-PMMA/THF and *st*-PMMA/toluene systems to characterize their HIC structures. In the profile of the *st*-PMMA/toluene gel, the diffraction peaks at *q*_tol,helix_ = 0.38 Å^−1^, *q*_helix,pitch_ = 0.78 Å^−1^ and *q*_helix,intra_ = 0.94 Å^−1^ result from the *st*-PMMA/toluene HICs, corresponding to the interhelical packing distance (*d*_tol,helix_ = 16.5 Å), helical pitch (*d*_helix,pitch_ = 8.0 Å) and intramolecular distance (*d*_helix,intra_ = 6.7 Å) along the helical axis, respectively (Kawauchi *et al.*, 2011 ▸). By contrast, the WAXS profile of the *st*-PMMA/THF solution exhibits two broad amorphous halos at *q*_hc,inter_ = 0.48 Å^−1^ and *q*_hc,intra_ = 0.89 Å^−1^, which correspond to the interchain (*d*_hc,inter_ = 13.1 Å) and intrachain (*d*_hc,intra_ = 7.0 Å) distances in the amorphous helical clusters of *st*-PMMA chains. This result suggests that the HICs were not formed in THF because the weak interaction between *st*-PMMA and THF induced spontaneous aggregation of the *st*-PMMA chains into small, helical-rich amorphous clusters [bottom model in Fig. 1 ▸(*b*)]. Interestingly, in the *st*-PMMA/toluene gel, although a portion of the helical *st*-PMMA chains were wrapped around toluene, forming HICs, the scattering peak at *q*_hc,inter_ indicated that a fraction of amorphous helical chains remained [Fig. 1 ▸(*b*)].

The helical conformation of *st*-PMMA primarily consists of *trans*–*trans* (*tt*) conformations (Spéváček, 1978 ▸; Berghmans *et al.*, 1994 ▸). As depicted in Fig. 1 ▸(*c*), the IR spectra of both *st*-PMMA/THF and *st*-PMMA/toluene exhibit CH_2_ rocking bands in the 840–870 cm^−1^ range, corresponding to the *trans*–*trans* (*ν*_*tt*_ ≃ 860 cm^−1^) and *trans*–*gauche* (*ν*_*tg*_ ≃ 841 cm^−1^) CH_2_ vibration modes. The *I*_860_/*I*_841_ ratio was used to assess the extent of helical conformation in *st*-PMMA (Berghmans *et al.*, 1994 ▸). In THF, helical *st*-PMMA exhibited a lower *I*_860_/*I*_841_ ratio of 0.69, which increased to 0.91 in the toluene solution. This result indicated that HIC formation promoted a more ordered helical conformation of the *st*-PMMA chains.

### 3.2. Gelation behavior of *st*-PMMA/toluene system

**Figure 2:**
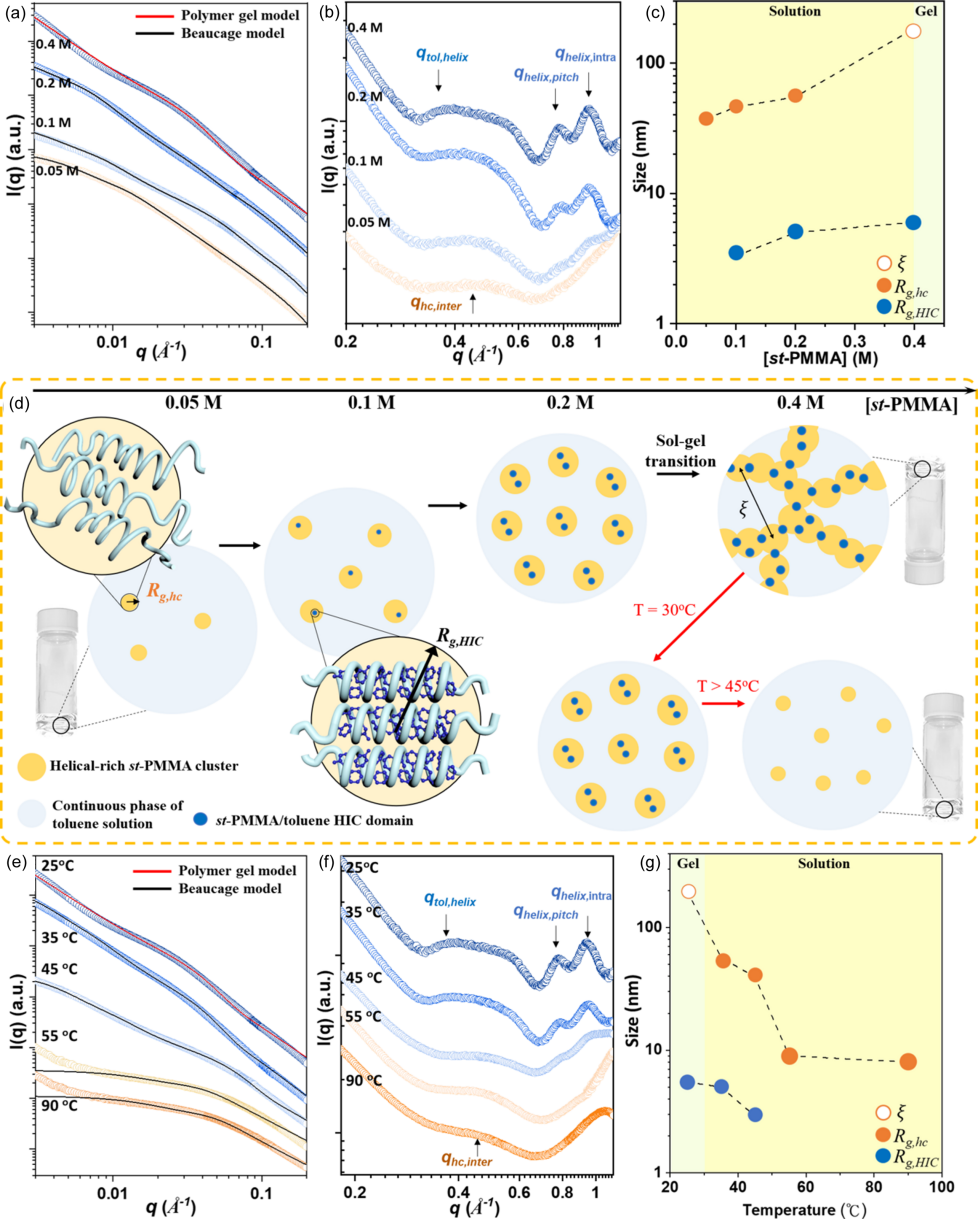
(*a*) SAXS and (*b*) WAXS profiles of *st*-PMMA/toluene solution for [*st*-PMMA] from 0.05 to 0.4 *M*. (*c*) Concentration-dependent variation of structural parameters (ξ, *R*_g,hc_ and *R*_g,HIC_) derived from SAXS model fitting in (*a*). (*d*) Illustration of structural evolution in the reversible sol-to-gel transition of the *st*-PMMA/toluene system. Temperature-dependent (*e*) SAXS and (*f*) WAXS profiles of *st*-PMMA/toluene at [*st*-PMMA] = 0.4 *M*. (*g*) Variation of ξ, *R*_g,hc_ and *R*_g,HIC_ derived from the SAXS model fitting in (*e*) during the gel-to-sol transition process.

Studies have demonstrated that *st*-PMMA readily forms HICs with aromatic solvents (Spéváček, 1978 ▸; Berghmans *et al.*, 1994 ▸). Therefore, it is essential to explore the thermodynamically favored structure during gelation of the *st*-PMMA/toluene system. An inverted vial test with varying [*st*-PMMA] concentrations in toluene solution (Fig. S3) indicated that the sol-to-gel transition occurred when the [*st*-PMMA] concentration reached 0.4 *M*, highlighting a strong correlation between the gelation behavior and self-assembly of HIC structures. To probe the structural evolution under varying [*st*-PMMA] concentrations, simultaneous SAXS/WAXS measurements were conducted, as illustrated in Figs. 2 ▸(*a*) and 2 ▸(*b*) (Liu *et al.*, 2019 ▸). To analyze the hierarchical structures within this two-component system, the SAXS profiles of the sol and gel states were fitted using the Beaucage and gel-like models, respectively (details provided in the supporting information) (Mallam *et al.*, 1991 ▸; Shibayama *et al.*, 1992 ▸; Beaucage, 1995 ▸). Fig. 2 ▸(*c*) presents the structural parameters derived from the SAXS analyses, and Fig. 2 ▸(*d*) illustrates the structural evolution during a reversible sol-to-gel transition in the *st*-PMMA/toluene system.

At the lowest [*st*-PMMA] of 0.05 *M*, only weak scattering halos corresponding to the disordered *st*-PMMA chains within the helical-rich clusters appeared in the WAXS region (*q* = 0.2–1.1 Å^−1^). In the SAXS region (*q* = 0.003–0.2 Å^−1^), the SAXS profiles in Fig. 2 ▸(*a*) exhibit a single scattering knee, which was attributed to the mesomorphic helical-rich *st*-PMMA clusters with a gyration radius (*R*_g,hc_) of 32 nm. As [*st*-PMMA] increased from 0.05 to 0.2 *M*, the intensity of *q*_hc,inter_ increased gradually [Fig. 2 ▸(*b*)], indicating an increase in the quantity of amorphous *st*-PMMA clusters in the solution. When [*st*-PMMA] exceeded 0.1 *M*, the diffraction peaks (*q*_tol,helix_, *q*_helix,pitch_ and *q*_helix,intra_) became evident, indicating gradual crystallization of the *st*-PMMA/toluene HICs within the helical-rich clusters. Moreover, the SAXS profiles [Fig. 2 ▸(*a*)] exhibited two scattering knees: one in the low-*q* region (*q* < 0.02 Å^−1^) and the other in the higher-*q* region (0.02 < *q* < 0.2 Å^−1^). These knees corresponded to the larger helical-rich clusters (*R*_g,hc_) and smaller helical bundles of *st*-PMMA/toluene HICs (*R*_g,HIC_), respectively, as fitted using the Beaucage model. Shifting of these scattering knees toward lower *q* values with increasing [*st*-PMMA] reflected the growth of *R*_g,hc_ and *R*_g,HIC_, as depicted in Fig. 2 ▸(*c*).

At [*st*-PMMA] = 0.4 *M*, gel formation occurred as mesomorphic helical-rich clusters collided and formed a larger-scale 3D network structure [Fig. 2 ▸(*d*)]. The diffraction peaks of *st*-PMMA/toluene HICs became prominent, while the peak intensity of *q*_hc,inter_ decreased, indicating a higher HIC content within the gel network [Fig. 2 ▸(*b*)]. On the basis of the gel-like model fitting, the correlation length (ξ) of the network structure and *R*_g,HIC_ were determined to be 185 and 5.9 nm, respectively. Fig. 2 ▸(*c*) summarizes the hierarchical sizes across the solution and gel states for various [*st*-PMMA]. From sol to gel, the larger domain (*R*_g,hc_ and ξ) grew from 55 to 185 nm, while *R*_g,HIC_ increased from 5 to 5.9 nm. Crystallization of the *st*-PMMA/toluene HICs occurred hierarchically within the pre-existing large helical-rich clusters, which lowered the nucleation barrier considerably. This process resembles a two-step crystallization (Chuang *et al.*, 2011 ▸), where conformational and concentration fluctuations induce phase-separated domains, facilitating subsequent crystallization. Thus, the helical-rich *st*-PMMA clusters act as intermediates for forming thermodynamically favored *st*-PMMA/toluene HICs.

To confirm the thermal stability of the *st*-PMMA/toluene HICs, temperature-dependent SAXS/WAXS profiles of the *st*-PMMA/toluene gel were obtained at [*st*-PMMA] = 0.4 *M*, as illustrated in Figs. 2 ▸(*e*) and 2 ▸(*f*). During heating, the diffraction peaks *q*_tol,helix_, *q*_helix,pitch_ and *q*_helix,intra_ diminished gradually and they disappeared at temperatures exceeding 45°C, leaving only the *q*_hc,inter_ scattering peak. Meanwhile, the SAXS profiles were reduced to a single scattering knee with reduced intensity at high *q*. Fig. 2 ▸(*g*) presents the size changes (ξ, *R*_g,hc_ and *R*_g,HIC_) derived from model fitting. The gel-to-sol transition occurred at around 35°C, while the disassembly temperature of the *st*-PMMA/toluene HICs (*T*_m,tolHIC_) is approximately 50°C. Additionally, these phase transitions were confirmed by performing inverted vial tests (Fig. S4) and recording temperature-dependent IR spectra (Fig. S5). The results indicated that the *st*-PMMA/toluene HICs played a critical role in the reversible sol-to-gel transition, as illustrated in Fig. 2 ▸(*d*).

### 3.3. Formation of *st*-PMMA/C_60_ HICs along temperature-controlled pathways

In supramolecular host–guest systems involving multiple guest molecules (Zwaag *et al.*, 2015 ▸; Valera *et al.*, 2018 ▸), the formation of various self-assembled structures is influenced heavily by the competition between the guest components. Consequently, in the *st*-PMMA/C_60_/toluene system, the *st*-PMMA host can form HICs with both C_60_ and toluene molecules (Yashima *et al.*, 2016 ▸; Berghmans *et al.*, 1994 ▸). Therefore, identifying the pathways to the thermodynamically favorable HICs is crucial for developing applications of the *st*-PMMA/C_60_/toluene system. Herein, we first prepared *st*-PMMA/C_60_/toluene samples with varying [*st*-PMMA] from 0.05 to 0.4 *M* while maintaining a constant C_60_ mixing ratio of 7 wt% relative to [*st*-PMMA]. This setup allowed us to create a competitive environment for comparing the binding affinities of both C_60_ and toluene to the *st*-PMMA host.

**Figure 3:**
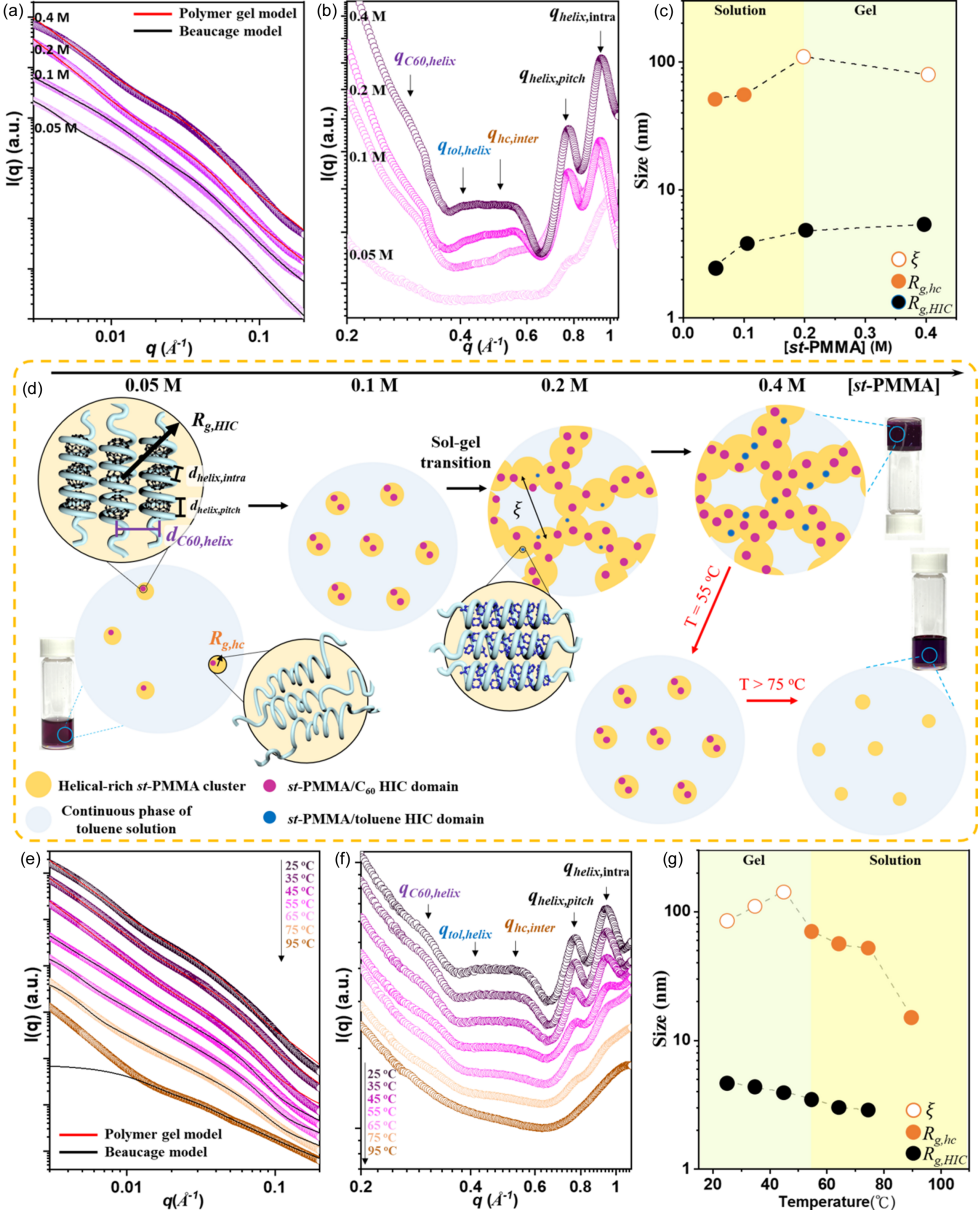
(*a*) SAXS and (*b*) WAXS profiles of the *st*-PMMA/C_60_/toluene system for [*st*-PMMA] of 0.05–0.4 *M*. (*c*) Variation of ξ, *R*_g,hc_ and *R*_g,HIC_ derived from the SAXS model fitting in (*a*). (*d*) Illustration of structural evolution in the *st*-PMMA/C_60_/toluene complex system. Temperature-dependent (*e*) SAXS and (*f*) WAXS profiles of the *st*-PMMA/C_60_/toluene system at [*st*-PMMA] = 0.4 *M*. (*g*) Variation of ξ, *R*_g,hc_ and *R*_g,HIC_ derived from SAXS model fitting in (*e*) during the gel-to-sol transition.

Figs. 3 ▸(*a*) and 3 ▸(*b*) depict the SAXS and WAXS profiles of the *st*-PMMA/C_60_/toluene system with various [*st*-PMMA]. Similarly to the *st*-PMMA/toluene system (Fig. 2 ▸), the two-level structure was characterized by ξ, *R*_g,hc_ and *R*_g,HIC_ determined through fittings using the Beaucage and gel-like models for the *st*-PMMA/C_60_/toluene system, as depicted in Fig. 3 ▸(*c*). The diffraction peak (*q*_C60,helix_) at *q* ≃ 0.30 Å^−1^, depicted in Fig. 3 ▸(*b*), grew gradually as [*st*-PMMA] increased, together with the above-mentioned peaks of the helical-rich *st*-PMMA clusters and *st*-PMMA/toluene HICs. This *q*_C60,helix_ peak, indicative of the packing distance between the *st*-PMMA/C_60_ helices [as illustrated in Fig. 3 ▸(*d*)], was absent in the *st*-PMMA/toluene system [Fig. 2 ▸(*b*)], and it emerged only after the addition of C_60_. The packing distances (*d*_C60,helix_ = 20.9 Å) of the *st*-PMMA/C_60_ HICs were greater than those of the *st*-PMMA/toluene HICs (*d*_tol,helix_ = 16.5 Å) owing to the larger molecular size of C_60_. Furthermore, the *q*_C60,helix_ peak was observed at [*st*-PMMA] = 0.05 *M*, while the *q*_tol,helix_ peak of the *st*-PMMA/toluene HICs was observed at 0.2 *M*. Guest molecules with stronger binding affinity generally facilitate the formation of guest–host complexes at lower concentrations (Matulis *et al.*, 2005 ▸). Consequently, under these competitive conditions in the *st*-PMMA/C_60_/toluene system, the *st*-PMMA hosts preferentially assembled with C_60_ owing to their stronger binding affinity.

Furthermore, Fig. 3 ▸(*b*) shows that the diffraction intensities increased noticeably, which highlighted the enhanced crystallinity of both the HICs in the *st*-PMMA/C_60_/toluene system. This result aligned with the macroscopic results obtained in the inverted vial test (Fig. S6), where the critical gelation concentration for the *st*-PMMA/C_60_/toluene system was only [*st*-PMMA] = 0.2 *M*, which was half the concentration required for the *st*-PMMA/toluene system. However, *R*_g,HIC_ remained approximately 2–5 nm [Fig. 3 ▸(*c*)], similar to that of the *st*-PMMA/toluene system [Fig. 2 ▸(*c*)]. This implies that the nucleation density of the HICs dominated the gelation behavior in the *st*-PMMA/C_60_/toluene system. As [*st*-PMMA] further increased to 0.4 *M*, the corresponding length (ξ) of the gel network became smaller than that at 0.2 *M* [Fig. 3 ▸(*c*)], indicating increased network density. This phenomenon was also reflected in the rheological measurement results (Fig. S7), where the gel modulus (*G*′) of the *st*-PMMA/C_60_/toluene gel (*G*′ = 80 Pa) was higher than that of the *st*-PMMA/toluene gel (*G*′ = 20 Pa).

Figs. 3 ▸(*e*)–3(*g*) depict the temperature-dependent SAXS and WAXS profiles along with the fitted parameters. These profiles demonstrate the thermal stabilities of the *st*-PMMA/toluene and *st*-PMMA/C_60_ HICs during the gel-to-sol transition. As illustrated in Fig. 3 ▸(*g*), the correlation length of the gel network increased slightly with temperature owing to thermal expansion. Above 55°C, the gel network disintegrated, accompanied by a continuous decrease in the size of the HICs (*R*_g,HIC_). This gel-to-sol transition was further confirmed by the inverted vial test (Fig. S8). Interestingly, as the gel collapsed, the *q*_tol,helix_ peak disappeared, whereas the *q*_C60,helix_ peak remained visible until 75°C. This finding suggests that the melting temperature (*T*_m,C60HIC_) of the *st*-PMMA/C_60_ HICs was approximately 75°C, considerably higher than *T*_m,tolHIC_ of the metastable *st*-PMMA/toluene HICs (around 50°C). Therefore, the *st*-PMMA/C_60_ HICs emerged as the thermodynamically favorable species compared with the metastable *st*-PMMA/toluene HICs in the *st*-PMMA/C_60_/toluene system.

**Figure 4:**
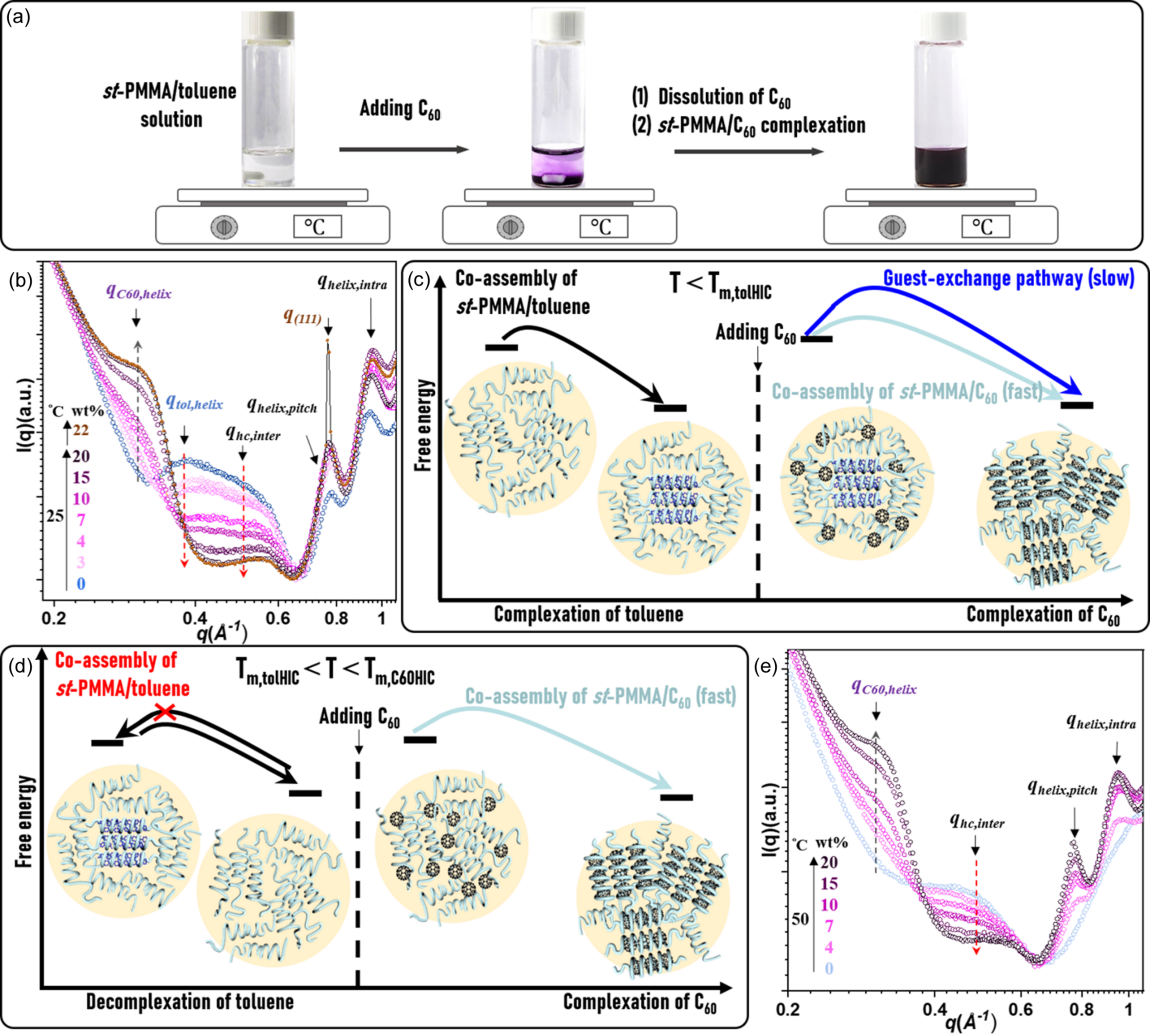
(*a*) Preparation of *st*-PMMA/C_60_ HICs in the *st*-PMMA/C_60_/toluene solution. (*b*) WAXS profiles of the *st*-PMMA/C_60_/toluene solution with various C_60_ wt% values at 25°C. (*c*) Free-energy landscape of the *st*-PMMA(0.4 *M*)/C_60_/toluene system at *T* < *T*_m,tolHIC_. (*d*) Free-energy landscape of the *st*-PMMA/C_60_/toluene system at *T*_m,tolHIC_ < *T* < *T*_m,C60HIC_. (*e*) WAXS profiles of the *st*-PMMA(0.4 *M*)/C_60_/toluene solution with various C_60_ wt% values at 50°C.

To further evaluate the maximum C_60_ encapsulation content in the *st*-PMMA host, Fig. 4 ▸(*a*) illustrates the procedure for preparing the *st*-PMMA/C_60_ HICs from the toluene solution at 25°C. On addition of C_60_ powder into the *st*-PMMA/toluene solution (0.4 *M*, 1 ml), the *st*-PMMA/C_60_ inclusion complexation drove the C_60_ powders to dissolve in the solution. We further used the WAXS tool to probe the pathways toward the *st*-PMMA/C_60_ HICs and determined the maximum C_60_ encapsulation ratio in the *st*-PMMA HICs at 25°C below *T*_m,tolHIC_. According to the WAXS profiles in Fig. 4 ▸(*b*), before adding C_60_, both the *st*-PMMA/toluene and the *st*-PMMA/C_60_ HICs coexist in the *st*-PMMA/toluene solution at 25°C below *T*_m,tolHIC_. As C_60_ was gradually added from 3 to 22 wt% (2.5–23 mg ml^−1^), the intensities of the *q*_tol,helix_ and *q*_hc,inter_ peaks decreased simultaneously. Meanwhile, the intensity of the *q*_C60,helix_ peak in the spectrum of the *st*-PMMA/C_60_ HIC increased. This observation shows that the *st*-PMMA/C_60_ HIC formation follows two self-assembly pathways at 25°C. In one of the pathways, the disordered *st*-PMMAs in the helical-rich cluster directly wrap around C_60_s to form the *st*-PMMA/C_60_ HICs. The other pathway is determined by competing encapsulation, where C_60_s undergoes a guest-exchange process with the *st*-PMMA/toluene HICs. As the C_60_ con­centration increases to 20 wt%, the disappearance of the *q*_tol,helix_ peak confirms that only the *st*-PMMA/C_60_ HICs remain in the system, as illustrated in Fig. 4 ▸(*c*). It is well known that C_60_ only has a limited solubility of approximately 1.5 mg ml^−1^ in toluene solvent (Guo *et al.*, 2016 ▸). Thus, *st*-PMMA acts not only as the helical host but also as the solubilizing agent to make C_60_ significantly more soluble in the *st*-PMMA/toluene solution through inclusion complexation. At 22 wt%, the sharp diffraction at *q*_(111)_ = 0.77 Å^−1^, attributed to the C_60_ crystallites, indicates that the C_60_ fills up the *st*-PMMA HICs, thereby leading to the precipitation of excess C_60_. From the thermodynamic standpoint, the maximum C_60_ encapsulation ratio in the *st*-PMMA helix with an *rr* content of 86% is approximately 20 wt%, lower than the ideal encapsulation ratio of 28 wt% in the defect-free *st*-PMMA helix (*rr* content = 100%) (Kajihara *et al.*, 2020 ▸). This decrease in encapsulation might be ascribed to a few chain defects in the *st*-PMMAs.

Nonetheless, as we follow the energy landscape at 25°C below *T*_m,tolHIC_ [Fig. 4 ▸(*c*)] to prepare the *st*-PMMA/C_60_HICs, a lengthy guest-exchange process lasting about 7.5 h is required to achieve an encapsulation ratio of 20 wt% (Mukhopadhyay *et al.*, 2006 ▸). Given that the free energy of the system is temperature dependent, temperature can modulate the energy landscape to bypass the guest-exchange process, as illustrated in Fig. 4 ▸(*d*). The WAXS analysis in Fig. 4 ▸(*e*) reveals that, at *T* = 50°C, within the range *T*_m,tolHIC_ < *T* < *T*_m,C60HIC_, the *st*-PMMA/toluene HICs disassemble as evidenced by the absence of their diffraction peaks (*q*_tol,helix_, *q*_helix,pitch_ and *q*_helix,intra_). As we added more C_60_s (from 4 to 20 wt%), the increased *I*_C60,helix_ at *q* = 0.30 Å^−1^ was accompanied only by the decreased *I*_hc,inter_ at *q* = 0.48 Å^−1^. This result clearly indicates that, at *T*_m,tolHIC_ < *T* < *T*_m,C60HIC_, the complex system directly chose the disordered *st*-PMMA/C_60_ co-assembly pathway to form the *st*-PMMA/C_60_ HICs, without undergoing the time-consuming guest-exchange process. This accelerated the formation of the *st*-PMMA/C_60_ HICs at the encapsulation ratio of 20 wt% in a shorter time of 4.5 h. This adjustment in temperature successfully tuned the energy landscape of the *st*-PMMA multicomponent system, thereby programming the efficient self-assembly route toward the thermodynamically favorable *st*-PMMA/C_60_ HICs.

### 3.4. *st*-PMMA/C_60_ HICs as redox sites for preparing the Ag SERS-active substrate

In sensing applications, numerous studies have demonstrated that the SERS effect in Raman spectroscopy depends on surface plasmons to enhance the light’s electric field, which is influenced by the morphology of metallic hotspots, including their size and density (Lee *et al.*, 2008 ▸; Zhu *et al.*, 2016 ▸; Solis *et al.*, 2017 ▸). C_60_ exhibits redox activity with specific metal ions, such as Ag and Au, through electron-transfer processes (Shin *et al.*, 2010 ▸; Shrestha *et al.*, 2013 ▸). However, the crystallization behavior of C_60_ often leads to micrometre- to millimetre-sized crystal morphologies, limiting the surface area available for metal ion adsorption (Wu *et al.*, 2015 ▸). Conversely, according to the above structural analysis, *st*-PMMA/C_60_ HIC bundles are nanometre sized, thereby providing a considerably larger surface area. Furthermore, use of the efficient co-assembly pathway [Fig. 4 ▸(*d*)] allows for rapid control over the C_60_ encapsulation ratio within the *st*-PMMA complex structure. Consequently, the *st*-PMMA/C_60_ HICs hold substantial promise as redox templates for fabricating Ag SERS-active substrates.

**Figure 5:**
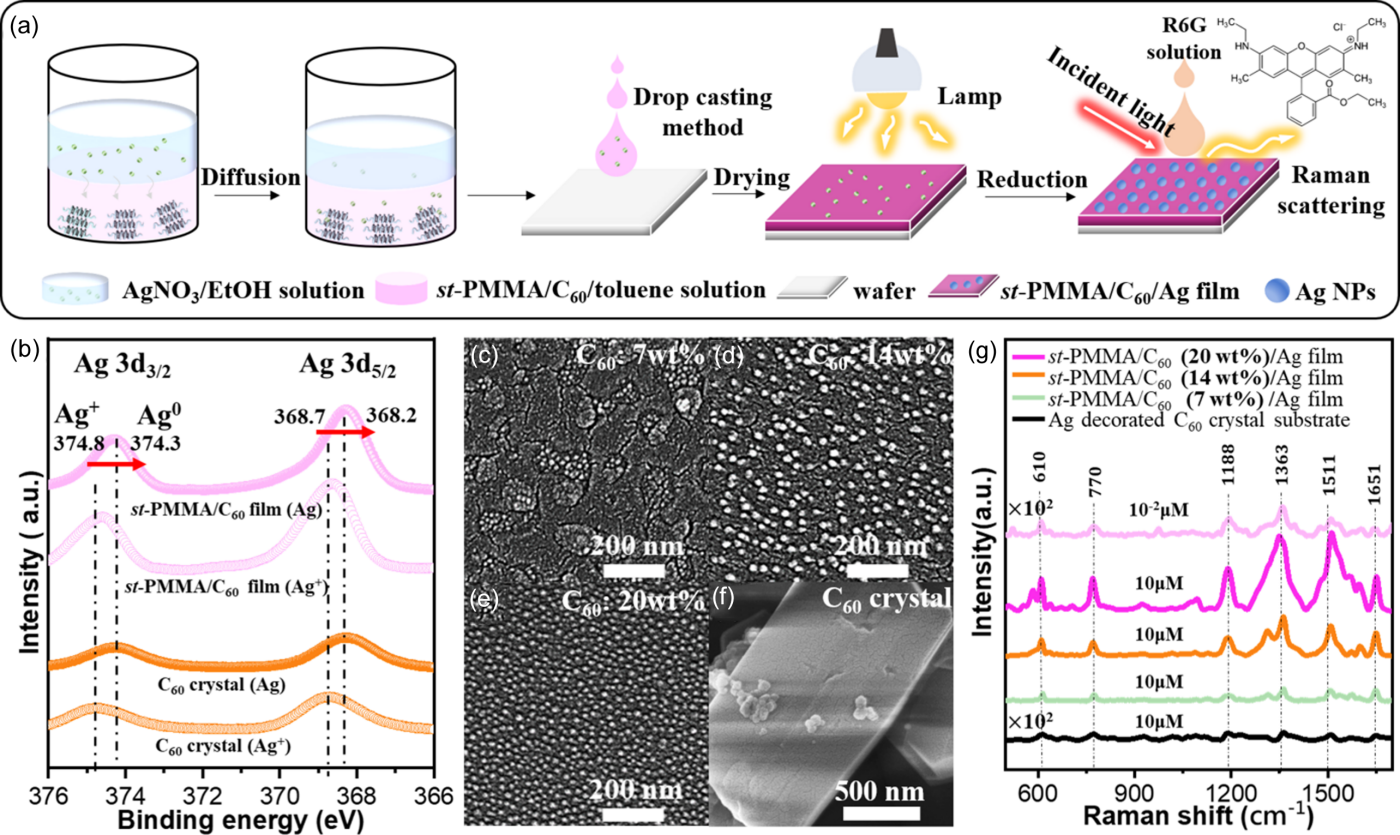
(*a*) Preparation of SERS-active Ag-NP substrates reduced from the *st*-PMMA/C_60_ complex film, and SERS measurement of R6G analytes on the Ag SERS-active substrate. (*b*) XPS spectra of the Ag^+^-containing *st*-PMMA/C_60_ HIC film and C_60_ crystals before/after visible light exposure. (*c*)–(*e*) SEM images of SERS-active Ag-NP substrates prepared with different C_60_ encapsulation ratios: (*c*) 7 wt%, (*d*) 14 wt% and (*e*) 20 wt%. (*f*) Ag-decorated C_60_ crystals. (*g*) Raman spectra (SERS) collected from various SERS-active Ag-NP substrates with different [R6G] (10 and 10^−2^ µ*M*).

The preparation procedure of the Ag SERS-active substrate is illustrated in Fig. 5 ▸(*a*). By using the liquid–liquid interface diffusion method (Shrestha *et al.*, 2013 ▸), a solution of AgNO_3_ in ethanol/H_2_O (2 ml) was gradually added to the *st*-PMMA/C_60_/toluene complex gel (1 ml) with various C_60_ encapsulation ratios (7, 14 and 20 wt%). This diffusion process yielded an Ag^+^-loaded *st*-PMMA complex gel that settled to the bottom of the vessel. After removing the supernatant, the Ag^+^-loaded *st*-PMMA complex gel was dissolved in a toluene solution and cast onto silicon wafers. The resulting Ag^+^-containing *st*-PMMA complex film was then subjected to a redox reaction under UV light exposure (λ = 365 nm) to obtain the Ag SERS-active substrate.

The redox process (Ag^+^ → Ag) within the *st*-PMMA/C_60_ HIC template was characterized using XPS. As depicted in Fig. S9, no XPS signals corresponding to Ag^+^/Ag were detected in the *st*-PMMA substrate, indicating minimal interaction between Ag^+^ and the *st*-PMMA host. However, Fig. 5 ▸(*b*) shows two distinct peaks at 368.7 and 374.8 eV, which correspond to the 3*d*_5/2_ and 3*d*_3/2_ binding energies of Ag^+^, respectively, in the *st*-PMMA/C_60_ HIC film and pure C_60_ crystal substrates. This suggests that Ag^+^ preferentially binds to C_60_ through charge-transfer interactions. Upon exposure to UV light, the binding energies of 3*d*_5/2_ and 3*d*_3/2_ shifted to lower values (368.2 and 374.3 eV, respectively), confirming successful redox conversion of the Ag^+^ into metallic Ag-NPs.

Moreover, SEM analysis revealed the distribution of metallic Ag-NPs on the redox templates. As depicted in Figs. 5 ▸(*c*)–5(*f*), Ag-NPs with an average size of approximately 20 nm were distributed evenly across the *st*-PMMA/C_60_ HIC films. As the C_60_ encapsulation ratio was increased from 7 to 20 wt%, the highest Ag-NP density was achieved on the *st*-PMMA/C_60_ (20 wt%) HIC film. By contrast, only a small number of Ag-NPs were found on the C_60_ crystal substrate. This difference was attributed to the uniformly dispersed C_60_ on the surface of the *st*-PMMA/C_60_ film, which provided numerous active sites that facilitated efficient Ag-NP reduction.

To evaluate the SERS performance of the Ag SERS-active substrates, aqueous solutions of rhodamine 6G (R6G, 10 µl) with varying concentrations (10^−2^–1 µ*M*) were applied to the substrates, as depicted in Fig. 5 ▸(*a*). The Raman spectra of R6G on the Ag-NP substrate, displayed in Fig. 5 ▸(*g*), clearly exhibit the vibrational bands of R6G in the 500–1700 cm^−1^ range (Jensen & Schatz, 2006 ▸). These bands were assigned as follows: the C—C—C in-plane bending band at ν = 610 cm^−1^, the C—H out-of-plane bending band at ν = 770 cm^−1^ and the C—H in-plane bending band at ν = 1188 cm^−1^. Additionally, the aromatic ring stretching bands of R6G were observed between ν = 1310 and 1649 cm^−1^. By contrast, no distinct Raman peaks of R6G (1 µ*M*) were detected on the Ag-decorated C_60_ crystal substrate [Fig. 5 ▸(*g*)].

The SERS enhancement factor (EF) of R6G on the Ag-NP substrates was calculated (see the supporting information). The substrates prepared from *st*-PMMA/C_60_ templates with encapsulation ratios of 7, 14 and 20 wt% exhibited EF_1512_ values of 3.1 × 10^4^, 1.5 × 10^5^ and 4.1 × 10^5^ at ν = 1512 cm^−1^, respectively. The increasing EF correlated to a higher density of Ag-NP formation on the templates with the greater C_60_ encapsulation. Furthermore, even at an [R6G] as low as 10x^−2^ µ*M*, clear Raman peaks were observed on the Ag-NP SERS substrate. These findings demonstrated that following an optimized self-assembly pathway facilitated the rapid formation of *st*-PMMA/C_60_ HIC films with the desired C_60_ encapsulation ratio. The well dispersed *st*-PMMA/C_60_ nanodomains allowed for the production of a higher number of Ag-NPs, leading to significant SERS enhancement.

## 4. Conclusions

This study elucidated the self-assembly pathways of the *st*-PMMA/C_60_/toluene system. According to our structural characterization results, *st*-PMMA exhibited stronger binding affinity for C_60_ than for toluene, making *st*-PMMA/C_60_ HICs the thermodynamically favored structures. Temperature control helped to modulate the energy landscapes of the self-assembled HICs: at *T* < *T*_m,tolHIC_, the complex guest-exchange pathway delayed *st*-PMMA/C_60_ HIC formation, whereas at *T*_m,tolHIC_ < *T* < *T*_m,C60HIC_, the *st*-PMMA/toluene HICs were suppressed, allowing for rapid *st*-PMMA/C_60_ HIC formation. The *st*-PMMA/C_60_ HIC film effectively acted as a redox template, generating abundant Ag-NPs for SERS applications. These substrates deliver a high EF of 10^5^, outperforming Ag-decorated fullerene crystals in R6G detection. This work provides a framework for programming self-assembly pathways to design advanced supramolecular materials.

## 5. Related literature

The following references are cited only in the supporting information: He *et al.* (2017 ▸); Wei & Hore (2021 ▸).

## Supplementary Material

Supporting figures. DOI: 10.1107/S1600576725001712/ge5169sup1.pdf

## Data Availability

Details of sample preparation, structural characterization methods, the fitting procedure and analyses are included in the supporting information.
