# Supplementary material for: Optimal st-PMMA/C60 helical inclusion complexes via tunable energy landscapes for the application of an Ag SERS-active substrate
Source: J Appl Crystallogr. 2025 Mar 19;58(Pt 2):553–63. doi: 10.1107/S1600576725001712 (PMC11957412; doi:10.1107/S1600576725001712)
Supplement: Supplementary file 1 [file j-58-00553-sup1.pdf]

JOURNAL OF APPLIED CRYSTALLOGRAPHY

Volume 58 (2025)

Supporting information for article:

Optimal *st*-PMMA/C60 helical inclusion complexes via tunable energy landscapes for the application of Ag SERS-active substrate

Song-Yu Tsai, Wen-Tsung Tseng, Jina-Hua Su, Yu-Hao Wang, Yi-Wei Chang, Chia-Hsin Wang, U-Ser Jeng, Kuan-Yi Wu, Chien-Lung Wang and Wei-Tsung Chuang

S1. Preparation of *st*-PMMA/C_{60}/toluene solution and gel

Approximately 10–80 mg of *st*-PMMA was first dissolved in 1.0 mL of toluene to obtain a final [*st*-PMMA] of 0.05–0.4M (10–80 mg.mL^{-1}), stirred at 110 °C for 30 min, and cooled to room temperature. C_{60} was dissolved in *st*-PMMA toluene solution under magnetic stirring. The C_{60} encapsulation ratio relative to *st*-PMMA was calculated as the amount of C_{60} in the toluene solution/(amount of C_{60} in the toluene solution + amount of *st*-PMMA in the toluene solution) by using 0 wt.% to 20 wt.% for the *st*-PMMA/C_{60}/toluene solution calculations. Any C_{60} in excess of 22 wt.% precipitates in the *st*-PMMA/C_{60}/toluene solution.

S2. Approximate modeling of hierarchical structure in *st*-PMMA multicomponent system

The experimental SAXS profiles presented in the manuscript were obtained by combining the scattering profiles contributed by the hierarchical structures of the larger helical clusters of *st*-PMMA chains and the smaller bundles of the *st*-PMMA helical inclusion complex (HIC). To visualize the structural hierarchy of the solution in the *st*-PMMA/toluene and *st*-PMMA/C_{60}/toluene solutions, the Beaucage model was utilized to model the hierarchical structures in the *st*-PMMA complex solution through decoupling of the scattering contributions at different structural levels by using the SASview software.(Beaucage, 1995, Wei, *et al.*, 2021) As expressed in Eq. S1, the Beaucage model indicates that the scattering intensity *I(q)* of the hierarchical self-assembled architecture is the summation of the form factors of the structures in *m* length scales.

$$\mathrm{I}(q) = G_1 \exp\left(-\frac{q^2 R_{g,HIC}^2}{3}\right) + G_2 \exp\left(-\frac{q^2 R_{g,hc}^2}{3}\right)\left(\frac{1}{q^*}\right)^P + B$$
$$q^* = q\left[erf\left(qR_{g,HIC}/\sqrt{6}\right)\right]^{-3} \qquad \textbf{Eq. (S1)}$$

where q^* is $q\left[erf\left(qR_{g,HIC}/\sqrt{6}\right)\right]^{-3}$; G_1 and G_2 are exponential prefactors; P and B denote a mass fractal index and the background, respectively.

Because helical *st*-PMMA clusters are found only in the dilute *st*-PMMA/toluene solution at [*st*-PMMA] = 0.05 M, the SAXS profile can be fitted using the Beaucage model with the structure level i = 1, which provides the $R_{g,hc}$ of the helical *st*-PMMA cluster, as expressed in Eq. S1. At higher [*st*-PMMA], the helical *st*-PMMA cluster and HICs (*st*-PMMA/toluene complex or *st*-PMMA/C_{60} complex) appear in the *st*-PMMA complex system. According to the simultaneous SAXS/WAXS analysis in the manuscript, the two self-assembled structures form a hierarchical structure in which the small domains of the *st*-PMMA HIC $R_{g,HIC}$ are embedded in the larger coiled *st*-PMMA

cluster ($R_{g,hc}$). Therefore, the Beaucage model with structural level $i = 2$ can be applied to conduct the modeling, as expressed in Eq. S1.

In addition, the *st*-PMMA/toluene and *st*-PMMA/C_{60}/toluene solutions can form gel structures above the critical gelation concentration. During the sol-gel transition, the helical clusters of *st*-PMMA chains merge to form a network structure, where the correlation length of the network heterogeneities can be defined. Furthermore, to maintain the structural integrity of the gel network, the smaller domains of the HICs act as junction points. To illustrate the hierarchical architecture of the physical gels, the gel-fitting model is given in Eq. S2 (Shibayama, *et al.*, 1992),

$$I(q) = \frac{I_L(0)}{(1+[(D+1)/3]Q^2\,\xi^2)^{D/2}} + I_R(0) \cdot \exp(Q^2\, R_{g,HIC}{}^2/3) \qquad \textbf{Eq. (S2)}$$

where D denotes the fractal dimension, ξ is the correlation length of the network heterogeneities in the gel network, and R_g represents the size of the smaller domains of the HICs. $I_L(0)$ and $I_R(0)$ denote the scattering factors that describe the scattering contribution of the network structure and the smaller HIC domains, respectively.

S3. Rheological measurement

The rheological properties of the *st*-PMMA/toluene and st-PMMA/C_{60} (7 wt.%)/toluene gels at [*st*-PMMA] = 0.4 M at 25 °C were measured using a rheometer (Anton Paar, MCR 302) equipped with a parallel plate. The shear rate was 0.0063 rad s^{-1}. The results are presented in Fig. S7.

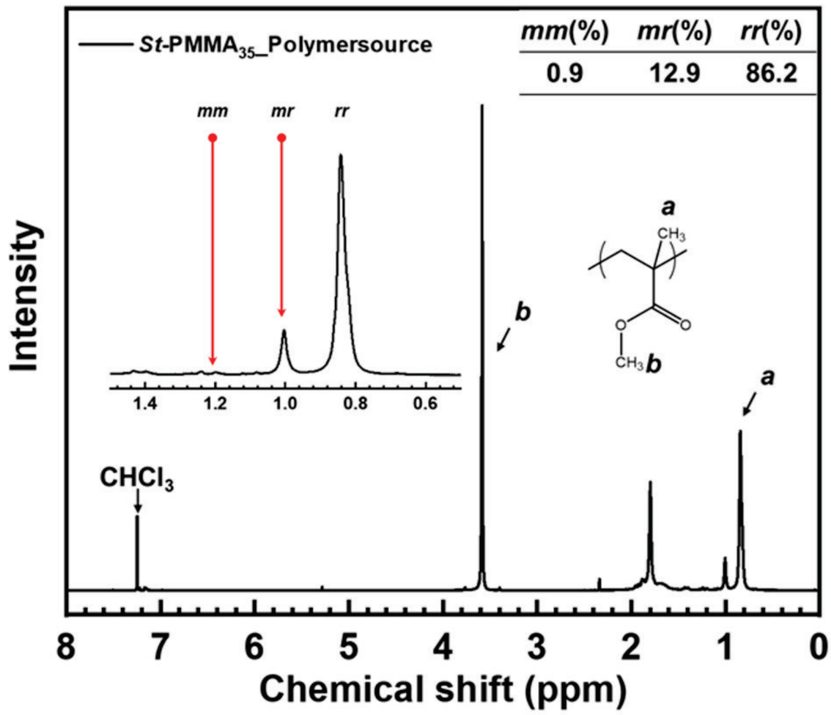

Figure S1 H[1] NMR spectra of *st*-PMMA with *rr* content of 86%. The spectra were measured in $CDCl_3$ at 25 °C.

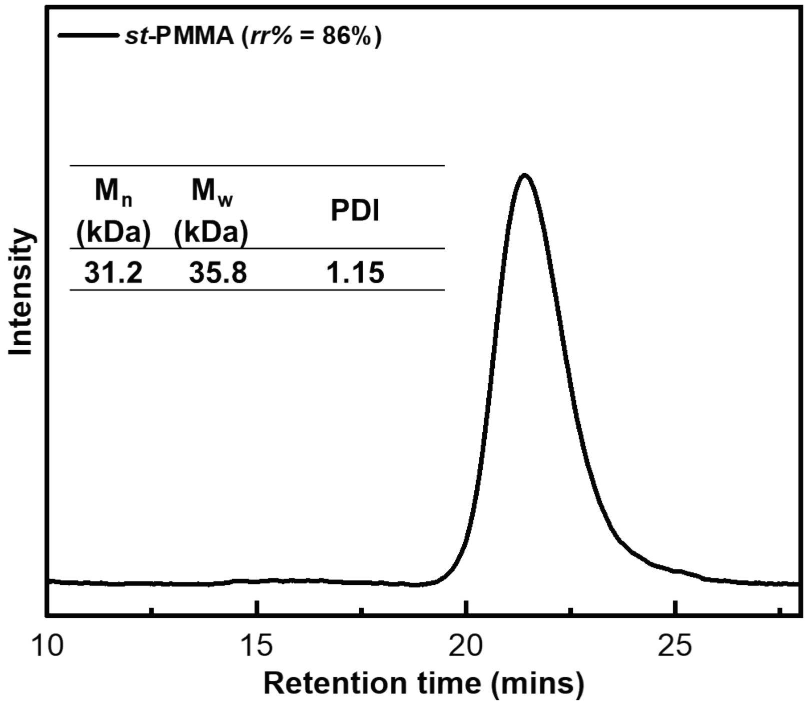

Figure S2 Gel permeation chromatography (GPC) analysis of *st*-PMMA with *rr* content = 86 %.

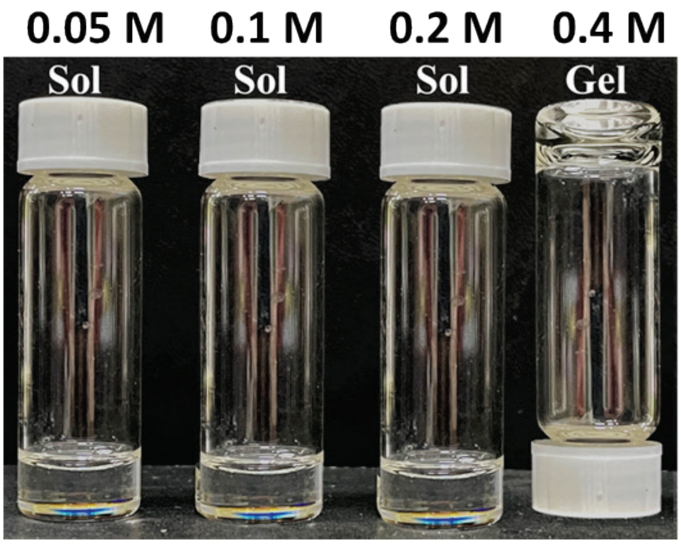

Figure S3 Series photos to capture the sol-gel transition of the *st*-PMMA/toluene solution as a function of [*st*-PMMA] from 0.05 M (10 mg.mL^{-1}) to 0.4 M (80 mg.mL^{-1}) at 25 °C.

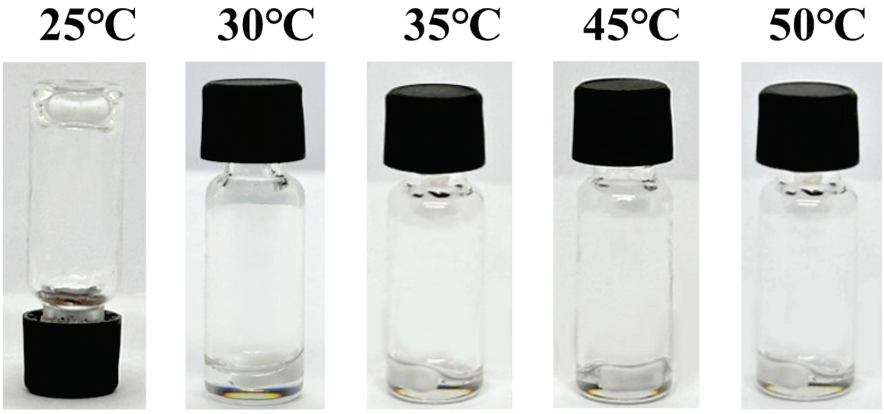

Figure S4 Series photos to capture the gel-sol transition of the *st*-PMMA/toluene solution with [*st*-PMMA] = 0.4 M in the temperature range of 25 °C–50 °C.

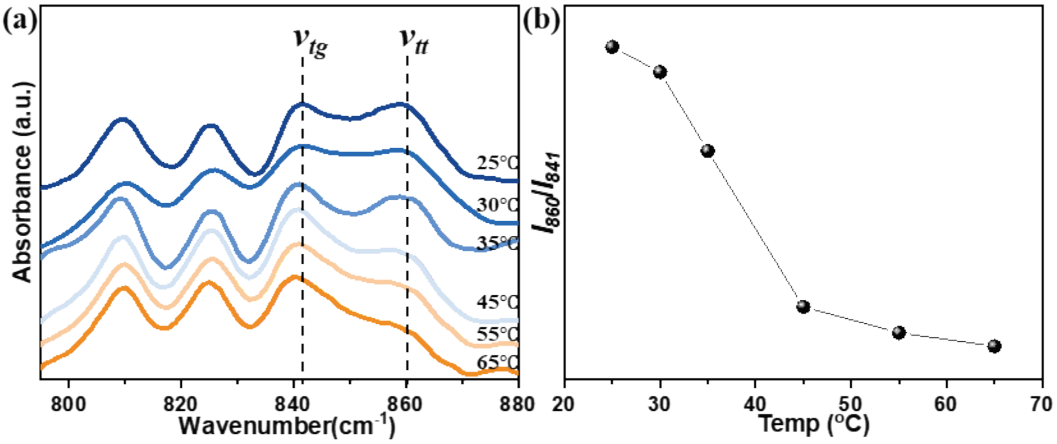

Figure S5 (*a*) Temperature-dependent IR spectrum of the *st*-PMMA/toluene solution from 25 °C to 65°C. (*b*) I_{860}/I_{841} ratio as a function of temperature calculated from (a) IR spectrum.

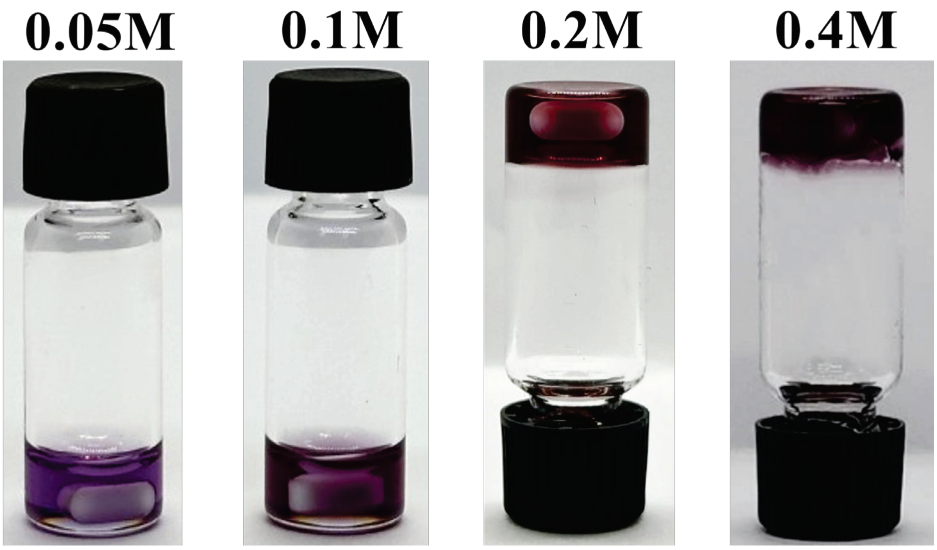

Figure S6 Series photos to capture the sol-gel transition of the *st*-PMMA/C_{60}(7 wt.%)/toluene solution as a function of [*st*-PMMA] from 0.05 M (10 mg.mL^{-1}) to 0.4 M (80 mg.mL^{-1}) at 25 °C.

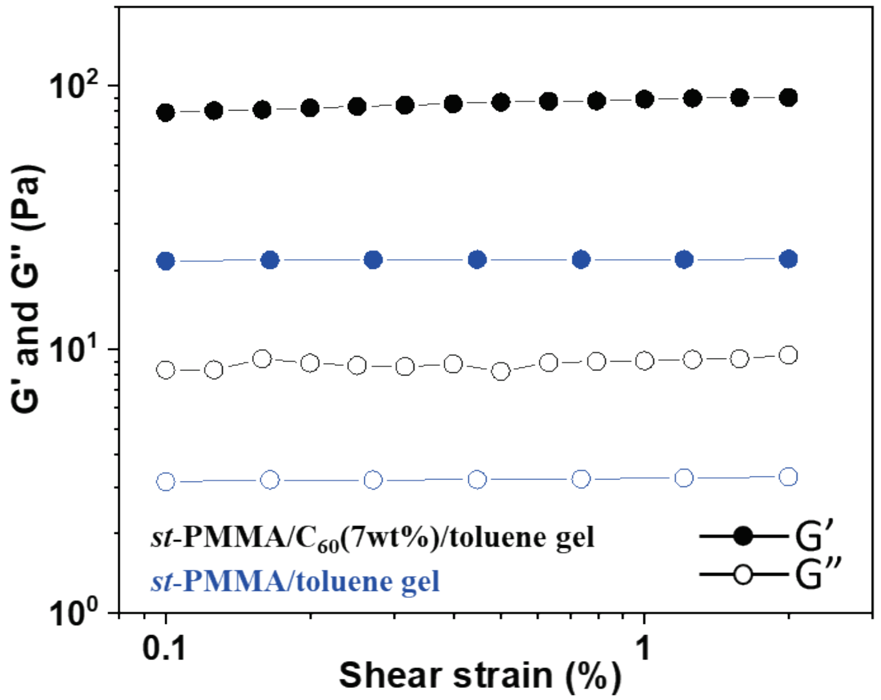

Figure S7 Relationship of storage modulus (G′, solid-circle line) and loss modulus (G″, hollow-circle line) with strain amplitude for *st*-PMMA/toluene gel (blue) and *st*-PMMA/C_{60}(7 wt.%)/toluene gel (black) at [*st*-PMMA] = 0.4 M (80mg mL^{-1}) and T = 25 °C.

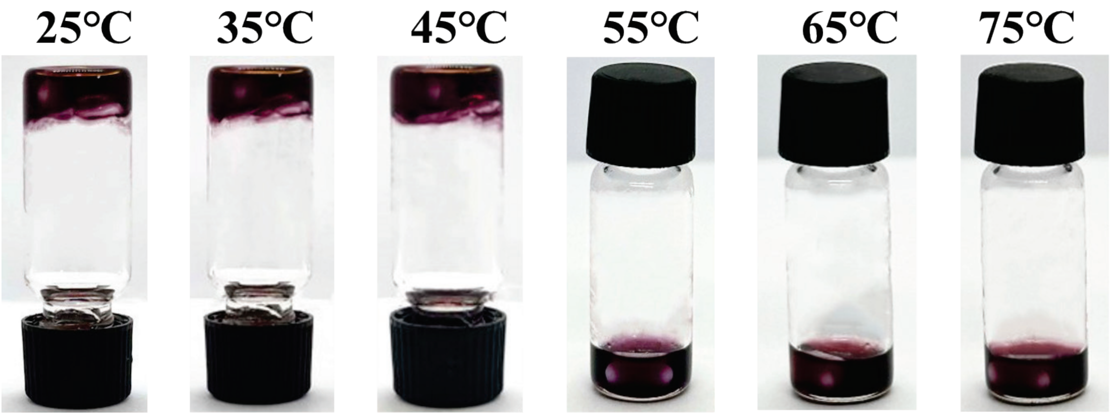

Figure S8 Series photos to capture the gel-sol transition of the *st*-PMMA/C_{60}(7 wt.%)/toluene solution with [*st*-PMMA] = 0.4 M in the temperature range of 25 °C–75 °C.

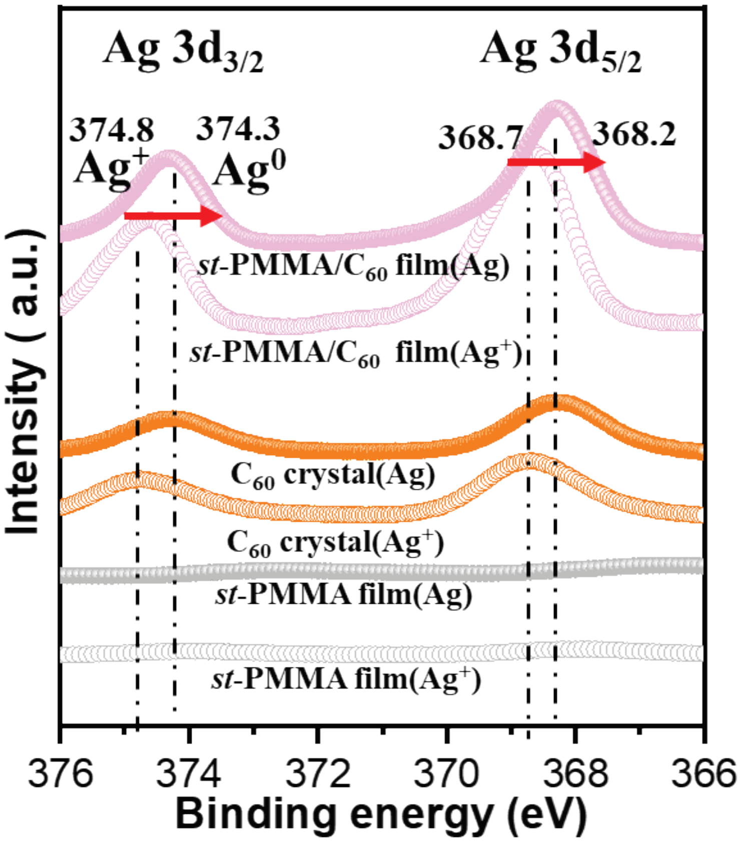

Figure S9 XPS spectra of Ag^+-containing *st*-PMMA/C_{60} complex film (magenta), C_{60} crystals (orange), and *st*-PMMA film (grey) before/after visible light exposure.

S4. Calculating SERS enhancement factor of R6G analytes on *st*-PMMA/C_{60}/Ag-NP substrate

The SERS enhancement factor (EF) of the R6Gs on the *st*-PMMA/C_{60}/Ag-NP substrates was calculated using the following equation:(He, *et al.*, 2017)

$$\text{E.F.} = \frac{I_{SERS}}{I_{Raman}} \times \frac{N_{Raman}}{N_{SERS}} \qquad \textbf{Eq. S3}$$

where I_{SERS} and I_{Raman} denote the intensity of the vibration bands of the R6G analyte under the SERS enhancement measurement on the substrates and normal Raman analysis, respectively. As depicted in Fig. S10, the vibration band at $\nu = 1512\ cm^{-1}$ exhibited a strong intensity and was selected for EF calculation. N_{SERS} and N_{Raman} denote the molecular numbers of the R6Gs under the spot size of the laser in the SERS measurement and Raman analysis. In the normal Raman analysis, $R6G_{(aq)}$ with a higher [R6G] = 10^{-1} M was dropped on the *st*-PMMA substrate. In the SERS analysis, $R6G_{(aq)}$ with a lower [R6G] = 10^{-5} M was dropped on the *st*-PMMA/C_{60}/Ag-NPs substrate.

Because the ratio of concentration difference was of the order of 10^4, the value of N_{Raman}/N_{SERS} changed to 10^{-4}. Furthermore, sample preparation on the substrates proceeded as follows: 10 μL of $R6G_{(aq)}$ was placed on the Ag-based SERS substrates. These substrates were air-dried at room temperature and then at 80 °C for 4 h. According to Eq. S4, the SERS enhancement factor of the R6Gs on the st-PMMA/C_{60}/Ag-NP substrates with various wt.% of C_{60} (7, 14, and 20 wt.%) were $EF_{1512} = 3.1 \times 10^4, 1.5 \times 10^5$ and 4.1×10^5 at ν = 1512 cm^{-1}, respectively.

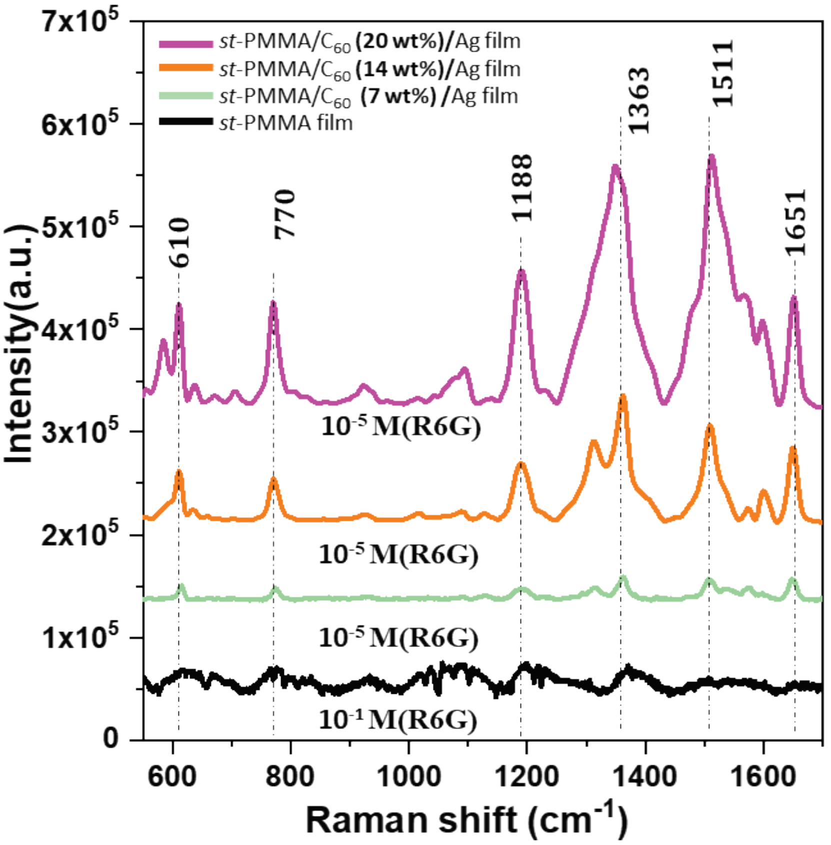

Figure S10 SERS spectra with 633-nm laser excitation of R6G molecules with 10^{-1} M on pure *st*-PMMA substrate (black) and 10^{-5} M on *st*-PMMA/C_{60}/Ag-NP substrates with 7 wt.% (green), 14 wt.% (orange), and 20 wt.% (magenta) of C_{60}.

Reference

Beaucage, G.,(1995) *J. Appl. Crystallogr.*, **28**, 717-728.

Wei, Y.; Hore, M. J. A.,(2021) *J. Appl. Phys.*, **129**.

Shibayama, M.; Tanaka, T.; Han, C. C.,(1992) *J. Chem. Phys.*, **97**, 6829-6841.

He, S.; Chua, J.; Tan, E. K. M.; Kah, J. C. Y.,(2017) *RSC adv.*, **7**, 16264-16272.
